# Welding fume exposure and prevalence of chronic respiratory symptoms among welders in micro- and small-scale enterprise in Akaki Kality sub-city, Addis Ababa, Ethiopia: a comparative cross-sectional study

**DOI:** 10.1186/s12890-024-02958-2

**Published:** 2024-03-20

**Authors:** Hager Badima, Abera Kumie, Bereket Meskele, Samson Wakuma Abaya

**Affiliations:** 1 Addis Ababa City Administration Health Bureau, Addis Ababa, Ethiopia; 2 Department Preventive Medicine, School of Public Health, Addis Ababa University, Addis Ababa,, Ethiopia

**Keywords:** Welding fume, Chronic respiratory symptoms, Associated factors, Welders

## Abstract

**Background:**

Exposure to welding fumes can lead to different respiratory health disorders, including lung cancer, due to long-term exposures. In Ethiopia, large numbers of people are engaged in the welding sector. Often, these workers are exposed to welding fumes at their workplaces, however, the level of exposure and its health effects have never been studied.

**Objective:**

To measure the level of personal welding fume exposure and assess chronic respiratory symptoms and associated factors, among micro and small-scale enterprise metal workshop workers, in Akaki Kality Sub city, Ethiopia.

**Methods:**

A comparative cross-sectional study involving 226 welders and 217 controls. Chronic respiratory symptoms were assessed using a standardized questionnaire adopted from the American Thoracic Society (ATS). Welding fumes were collected from the welder’s breathing zone using 37 mm close-faced plastic cassettes fitted with Polyvinyl Chloride (PVC) filters connected to Casella pumps at an airflow rate of 2 L/min.

**Result:**

The overall prevalence of chronic respiratory symptoms among welders and controls were 54 (23.9%) and 20 (9.2%) respectively. The geometric mean and geometric standard deviation (GSD) of personal welding fume exposure, among welders was 5.98 mg/m^3^ (± GSD = 1.54). In this study, 53.3% of the samples exceeded the Occupational Exposure Limit defined by the American Conference of Governmental Industrial Hygiene. Chronic respiratory symptoms were significantly associated with educational status (Adjusted Odds Ratio (AOR): 5.11, 95% CI: 1.35, 19.33), respiratory protective equipment use (AOR: 3.33, 95% CI: 1.52, 7.31), safety training (AOR: 2.41, 95% CI: 1.10, 5.28), smoking (AOR:3.57, 95% CI: 1.54, 8.23), welding machine maintenance (AOR: 1.87, 95% CI: 1.01, 3.59) and welding site (i.e. indoors vs. outdoor) (AOR: 6.85. 95% CI: 2.36, 19.89).

**Conclusions:**

The prevalence of chronic respiratory symptoms among welding workers was significantly higher than controls. More than half of the samples exceeded the Occupational Exposure Limit. Educational status, implementation of safety training, and welding sites were significantly associated with chronic respiratory symptoms. The results suggested a need to reduce welding fume exposure to improve the respiratory health of the workers.

## Background

Welding is an industrial process whereby two metallic parts are heated to their melting point to be joined together. A third filler metal is included in the melting process to make the joint stronger. The fumes that are produced during this process contain different types of metal and gaseous substances [1]. Globally, welders account for more than 1% of the labor force. These workers are exposed to occupational welding fumes in their daily activities [2]. It is well-documented that exposure to welding fumes for a prolonged period causes metal fume fever, which gives flu-like dyspnea and cough-like symptoms [3]. In 2017, the International Agency for Research on Cancer (IARC) determined that agents produced by welding fumes are carcinogenic for human beings [4]. They are categorized as a group one cause of cancer [5]. Exposure to welding fumes and lung function have been shown to have a negative correlation [6].

A study conducted in Malaysia indicated that exposure to welding fumes can cause respiratory problems among welders including coughing, phlegm, chest illnesses, nausea, and fatigue [7]. A study from Macedonia indicated a higher prevalence of respiratory symptoms among welders, with the highest reported for coughing (73.3%) and phlegm (80.0%) [8]. Another study conducted in Saudi Arabia revealed a higher prevalence of respiratory complaints, notably chronic bronchitis, among welders compared to the non-exposed group [8]. This is attributed to the emissions generated during welding processes. In a study carried out in Kazakhstan’s metalworking industries, it was found that welders/assemblers exhibited markedly elevated levels of FeNO [9]. This suggests potential respiratory tract inflammation linked to the exposure to airborne at their workplace at their workplace [9].

Studies have indicated that the use of respiratory protective equipment, the presence of adequate ventilation during welding activity, and exposure level were major factors associated with chronic respiratory symptoms among welders [10, 11].

In Ethiopia, large numbers of people are engaged in the welding sector. These workers are exposed to welding fumes at their workplaces. However, the level of exposure and its health effects have never been studied. Therefore, this study aims to assess the personal welding fume exposure and prevalence of chronic respiratory symptoms and associated factors among welders in micro and small-scale enterprises in Akakai Kality sub-city Ethiopia.

## Methods

### Study area

This study was conducted in Micro and small-scale enterprise metal workshops found in the Akaki Kality sub-city, Addis Ababa, Ethiopia. Akaki kality is one of the 10 sub-cities found under the Addis Ababa city administration. Akaki Kality sub-city was selected because it has the largest number of micro and small-scale enterprise metal workshop establishments in the city. Additionally, the majority of the population in the sub-city relies heavily on industrial activities for their income. Akaki Kality sub-city has 13 districts. In the sub-city, there are a total of 91 micro and small-scale enterprise metal workshop establishments with a total of 658 workers as welders. Taking into consideration the available resources, we selected 3 districts randomly from thirteen sub-cities. There were 46 metal shops in these three districts. Office workers were used as a control because we assume they are not exposed to welding fumes or other dust at work.

### Study design, and period

A comparative cross-sectional study design was conducted among welders in metal shops and office workers in medium- and small-scale enterprises found in Akaki Kality Sub-cities, Addis Ababa, Ethiopia from September 01, 2020, to October 30, 2020.

### Sample size and sampling procedure

#### For chronic respiratory symptoms

The sample size for the prevalence of respiratory symptoms among welders was calculated with a double proportion formula considering the prevalence of respiratory symptoms among exposed and non-exposed 32% and 13.8% respectively [12] to obtain 85% statistical power for the detection of this difference in respiratory symptoms between the two groups, at a significance level of 0.05. After considering 10% for non-response, a total of 466 participants (i.e., 233 from welders and 233 from office workers) were enrolled in the study. The total sample size of 233 was proportionally distributed to all metal workshops located in the three districts. Then welders were selected by systematic random sampling method from each metal workshop, using the workers’ registration list as a sampling frame. Similarly, 233 office workers were allocated in proportion to the size of the thirteen medium and small enterprise offices found in the Akaki Kality Sub-city. The office workers were selected by systematic random sampling method from each medium and small enterprise office using the workers’ registration list as a sampling frame. All participants in the study were male, reflecting the male-dominated nature of the welding profession in Ethiopia.

### Personal welding fume exposure level

One metal workshop was randomly selected from each three districts. The sample size for personal welding fume exposure assessment from these metal workshops was based on Rappaport et al. 2008 recommendation that 5–10 randomly selected individuals in a Similar Exposure Group (SEG) with repeated measurements are sufficient to predict the group exposure level [13]. SEG refers to a group of workers working the same type of job in a similar work area for the same duration of time. With the above assumption there was only one SEG as these shops were medium- and small-scale enterprises. Five workers were randomly selected from each metal workshop. Thus, a total of 15 welders were involved in this study with repeated measurements a total of 30 welding fumes were collected.

### Data Collection

#### Interview-chronic respiratory symptoms interview

The chronic respiratory symptoms among participants were assessed with face-to-face interviews using a standardized structured questionnaire adapted from the American Thoracic Society (ATS) [14]. The standardized questionnaire includes socioeconomic-demographic factors, duration of exposure, previous occupational history, smoking habits, past history of respiratory illnesses, and respiratory symptoms such as cough, phlegm, wheezing, shortness of breath, and nose irritation, particularly those associated with the risk of respiratory morbidity.

### Welding fume exposure measurements

Full-shift personal welding fume was sampled in the workers’ breathing zone using a 37 mm diameter Millipore plastic sampling cassette fitted with polyvinyl carbonated (PVC) filters of 5 μm pore size connected to Side Kick Casella (SKC) pumps operating at 2 L/min [15]. The exposure measurements were conducted on randomly chosen days and repeated sampling was conducted the next day.

### Observational checklist

Observational checklists were used to check working conditions such as ventilation systems, types of welding materials used, types and appropriateness of PPE, and location of the welding area.

### Data quality assurance

For personal welding fume measurement, the airflow rate in the sampling pumps was measured and recorded before and after each sampling event using a Rota meter (i.e., flow rates more than ± 10% different from the target flow rate of 2.0 lit/min was dropped) [15]. At the end of sampling, the filter cassettes were covered and carefully handled in a labeled container to prevent damage and transported to the lab for analysis. Field blanks were used to correct for any weight changes during sampling.

For chronic respiratory symptoms, a standardized questionnaire was used to ensure data quality. Prior to data collection, training was given to data collectors and supervisors to fill out the questionnaire appropriately and to reduce bias. Additionally, the questionnaire was translated from English to Amharic and back to English using standard procedure to check its consistency. Each day the supervisor checked each questionnaire for completeness and consistency. In addition, pre-tests were carried out a week before the actual data collection to check the competency of the data collectors, and the reliability and validity of the data collection tools.

### Data management and analysis

The data were coded, and no names were included in the database. The results were described using arithmetic mean, geometric mean (GM), and geometric standard. The welding fume samples were analyzed gravimetrically using a standard microbalance scale AT261 Mettler Toledo with a detection limit of 0.01 mg m^− 3^ in the Environmental and Occupational Health laboratory at the College of Health Sciences of Addis Ababa University. The results were compared with the American Conference of Governmental Industrial Hygienists standard of 5 mg/m^3^ for welding fumes, measured as total particulates in the welder’s breathing zone [16].

Logistic regressions were used to identify factors associated with chronic respiratory symptoms. In the model, the chronic respiratory symptom was used as the dependent variable and socio-demographic, behavioral characteristics, administrative factors, and Environment factors were used as the independent variable. Chronic respiratory symptom was defined as the development of one or more of the symptoms of cough, Cough with sputum, breathlessness, and chest tightness which lasted at least three months in one year. Only variables with a P-value < 0.2 in the binary logistic analysis were transferred to multivariate analysis.

Adjusted Odds Ratio (AOR) with a 95% Confidence Interval (CI) was used to verify the association between the dependent and independent variables individually. The statistical significance level was set to a p-value less than 0.05. The analysis was done using the statistical software SPSS version 22.

## Results

### Characteristics of participants

A total of 443 (226 welders and 217 controls) were involved in this study. The mean age for welders was 29 (± SD = 6.796) and 28.58 (± SD = 4.909) for controls. There was a significant age difference between welders and office workers. The mean work experience of welders and controls was 5.47 (± SD = 5.166) and 4.25(± SD = 4.137) respectively. The majority of 128 (59%) of the welders claimed that they used a flame and fume-proof hand-held face shield (Table 1). The smoking status of the study participants showed that 31 (10.6%) welders and 23 (10.6%) controls were ever smokers (Current smokers and Ex-Smokers). Similarly, about 45 (20.7%) of the office workers were alcohol consumers and there was a significant difference in alcohol consumption between welders and office workers. As far as the workplace environment, most welders claimed they did their welding tasks both indoors and outdoors on most of their working days 90 (39.8%) (Table 1).

**Table 1:** Characteristics of welders and office workers in Akaki Kality sub-city, Addis Ababa, Ethiopia

Response	Welders	Controls	Total	P value
	n (%)	n (%)	n (%)	
Age Group				
Less than 25	80 (35.4)	57 (26.3)	137 (30.9)	0.038 ^*^
More than 25	146 (64.6)	160 (73.7)	306 (69.1)	
**Educational Status**				
Primary and secondary	216 (95.6)	28 (12.9)	244 (55.1)	< 0.001^*^
College Education	10 (4.4)	189 (87.1)	199 (44.9)	
**Work Experience**				
Less than 5 Years	142 (62.8)	155 (71.4)	297 (67)	0.376
More than 5 Years	84 (37.2)	62 (28.6)	146 (33)	
**Respiratory Protective Equipment used**				
Fume proof	123 (60.3)	N/A	123 (60.3)	
Non fume proof	81 (39.7)	N/A	81 (39.7)	
**Smoking Status**				
Ever Smoker	31 (10.6)	23 (10.6)	54 (12.2)	0.316
Non-Smoker	195 (86.5)	194 (89.4)	389 (87.8)	
**Alcohol Intake Status**				
Ever Consumer	88 (38.9)	45 (20.7)	133(30)	0.001^*^
Non-Consumer	138 (61.1)	172 (79.3)	310 (70)	
**Work-Related Medical Checkup**				
Yes	99 (43.8)	79 (36.4)	178 (40.2)	0.14
No	127 (56.2)	138 (63.6)	265 (59.8)	
**Working Hours in days**				
Up to 8 h	101 (44.7)	211 (97.1)	312 (79.4)	< 0.001^*^
More than 8 h	125 (55.3)	6 (4.6)	131 (29.6)	
**Safety Training**				
Yes	33 (15.5)	9 (4.3)	42 [10]	< 0.001^*^
No	180 (84.5)	199 (95.7)	379 (90)	
**Type of Welding Material**				
Shielded Metal Arc Work	216 (95.6)	N/A	216 (95.6)	
Gas Metal Arc Work	10 (4.4)	N/A	10 (4.4)	
**Welding Machine Calibration**				
Yes	87 (38.2)	N/A	87 (38.2)	
No	139 (61.8)	N/A	139 (61.8)	
**Welding Site**				
Outdoor	58 (25.7)	N/A	58 (25.7)	
Indoor	78 (34.5)	N/A	78 (34.5)	
Both	90 (39.8)	N/A	90 (39.8)	

*Note* n refers to number, % refers to percentage and N/A refers to not applicable; ^*^ statistically significant difference

### Personal total welding fume exposure

All of the study participants were male, ranging in age from 18 to 38 years. The overall personal total welding fume exposure ranged from 3.13 to 11.08 mg/m^3^(Table 2). The geometric mean of their personal welding fume exposure was 5.98 mg/m^3^(± GSD = 1.54) (Table 2). Among the 30 welding fume samples, 16 (53.3%) exceeded the occupational exposure limit which is 5 mg/m^3^ set by the American Conference of Governmental Industrial Hygienists (ACGIH) and the Occupational Safety and Health Administration (OSHA).

**Table 2:** Personal welding fume exposure levels among welders in Akaki Kality sub-city, Addis Ababa, Ethiopia

Establishment	Average welding fume Exposure (mg/m^3^)	Average Sampling time (minute)	Mean (± SD) (mg/m3)	GM (± GSD) (mg/m3)	Samples Exceeding ACGIH & OSHA (5 mg/m3) n (%)
Establishment 01	5.54	535	7.09 (± 3.38)	6.38 (± 1.67)	3 (60)
	4.53	536			
	11.075	512			
	3.555	492			
	10.775	537			
Establishment 02	8.895	510	5.11 (± 2.15)	4.76 (± 1.48)	1 [20]
	4.98	543			
	4.62	530			
	3.125	501			
	3.9	468			
Establishment 03	9.545	543	7.34 (± 2.37)	6.94 (± 1.45)	4 (80)
	9.365	536			
	4.245	542			
	8.345	542			
	5.205	523			
All establishments	3.13–11.08	468–543	6.51 (± 2.80)	5.98 (± 1.54)	16 (53.3)

*Note* n = number of samples (i.e., 15 welders participated in the study, with repeated measurements from each individual, resulting in a total of 30 welding fume samples); % = percentage; GM = geometric mean; GSD = geometric standard deviation; SD = *standard* deviation; ACGIH = American Conference of Governmental Industrial Hygiene; OSHA = the Occupational Safety and Health Administration

### Prevalence of chronic respiratory symptoms

The overall prevalence of chronic respiratory symptoms among welders and controls was 23.9% and 9.2% respectively (Table 3). The odds of developing a chronic respiratory symptom were higher among welders than control groups (AOR; 2.82; 95% CI; 1.51, 5.26) after adjusting for sex, educational status, monthly income, respiratory protective equipment utilization, alcohol intake, working hours in a day, working day in week, and safety training.

**Table 3:** Prevalence of chronic respiratory symptoms among metal and office workers in Akaki Kality sub-city Addis Ababa, Ethiopia

Response	Welders	Control	AOR (95%CI)	P value
	n (%)	n (%)		
Cough				
Yes	41 (18.1)	17 (7.8)	2.05 (1.06–3.96)	**0.032** ^*^
No	185 (81.9)	200 (92.2)		
Phlegm				
Yes	21 (9.3)	12 (5.5)	3.24 (1.20–8.17)	0.02*
No	205 (90.7)	205 (94.5)		
Breathlessness				
Yes	14 (6.2)	4 (1.8)	3.52 (1.14–10.85)	0.02*
No	212 (93.8)	212 (98.2)		
Chest tightness				
Yes	11 (4.9)	5 (2.3)	2.17 (0.74–6.35)	0.148
No	215 (95.1)	212 (97.7)		
Chronic Respiratory Symptoms				
Yes	54 (23.9)	20 (9.2)	2.82 (1.51–5.26)	**0.001** ^*^
No	172 (76.1)	197 (90.8)		

*Note* n = number of samples; % = percentage; AOR = Adjusted Odds Ratio; *P*-value for independent *t* test, *p* < 0.05; Chronic respiratory symptom was defined as the development of one or more of the symptoms of cough, Cough with sputum, breathlessness, and chest tightness which lasted at least three months in one year

### Factors associated with the occurrence of chronic respiratory symptoms among metal workers

The multivariate logistic regression was performed to identify factors associated with the occurrence of chronic respiratory symptoms among welders. Only variables with a P-value < 0.2 in the binary logistic analysis were transferred to multivariate analysis. Welders who were older than 25 years were 2 times more likely to develop chronic respiratory symptoms than those who were less than 25 years old (AOR: 2.12, 95%CI: 1.12, 4.46) (Table 4). Compared to welders who had college education, those who only had primary and secondary education were 5 times more likely to develop a chronic respiratory symptom (AOR: 5.11, 95% CI:1.35, 19.33). Welders who did not use protective respiratory equipment were 3 times more likely to develop chronic respiratory symptoms compared to those who used such equipment (AOR: 3.33, 95% CI: 1.52, 7.31). Welders who smoked were 3 times more likely to develop a chronic respiratory symptom than those who were nonsmokers (AOR: 3.57, 95%CI: 1.54, 8.23). Welders who were not given safety training were 2 times more likely to develop chronic respiratory symptoms than those who were given such training (AOR: 2.41, 95% CI: 1.10, 5.28). Welders who did not calibrate or maintain their welding machines were almost 2 times more likely to develop chronic respiratory symptoms than those who did not (AOR: 1.87, 95% CI: 1.01, 3.59). Compared to welders who undertook their welding tasks outdoors, welders who did their welding tasks indoors were 6 times more likely to develop chronic respiratory symptoms (AOR:6.85; 95% CI: 2.36, 19.89) (Table 4).

**Table 4:** Multivariate analysis for factors associated with the occurrence of chronic respiratory symptoms among metal welders, Akaki Kality sub-city, Addis Ababa, Ethiopia

Response	Chronic respiratory Symptoms		COR (95% CI)	AOR (95% CI)
	Yes	No		
Age Group				
less than 25	12	68	1	1
More than 25	42	104	2.29 (1.12–4.66)	2.12 (1.12–4.46)
Educational Status				
Primary and secondary	47	169	5.25 (1.42–19.36)	5.11 (1.35–19.33)
College Education	7	3	1	1
Work Experience				
Less than 5 Years	19	82	1	1
More than 5 Years	35	90	1.6 (0.89–3.16)	1.35 (0.69–2.63)
Respiratory Protective Equipment used				
Fume-proof	41	90	1	1
No-fume-proof	13	82	2.97 (1.45–6.03)	3.33 (1.52–7.31)
Smoking Status				
Smoker	16	15	4.41 (2.0–9.70)	3.57 (1.54–8.23)
Non-Smoker	38	157	1	1
Alcohol Intake Status				
Consumer	31	59	2.58 (1.38–4.82)	2.04 (1.04–3.99)
Non-Consumer	23	113	1	1
Work-Related Medical Checkups				
Yes	31	68	1	1
No	23	104	2.06 (1.11–3.83)	3.12 (1.50–6.48)
Working hours in a day				
Up to 8 h	19	82	1	1
more than 8 h	35	90	1.68 (0.89–3.16)	1.63 (0.88–3.18)
Safety training				
Yes	13	20	1	1
No	41	152	2.41 (1.11–5.25)	2.41 (1.10–5.28)
Welding machine calibration				
Yes	29	58	1	1
No	25	114	2.2 (1.18–4.11)	1.87 (1.01–3.59)
Welding Site				
Outdoors	19	38	1	1
Indoors	6	72	6.0 (2.21–16.29)	6.85 (2.36–19.89)
Both	29	62	1.06 (0.52–2.16)	1.13 (0.55–2.32)

COR = Crude odds ratio, AOR = Adjusted odds ratio, CI = Confidence Interval, 1.00 = reference; Adjusted for age, educational level, alcohol intake, PPE utilization, safety training, and welding site

## Discussion

The overall prevalence of respiratory symptoms among welders and controls was 23.9% and 9.2% respectively. The overall geometric mean value of the personal welding fume exposure among welders was 5.95 mg/m^3^. The multivariate modeling revealed that working indoors, regardless of smoking and age, was associated with an elevated risk of experiencing more respiratory symptoms. Conversely, the utilization of personal protective equipment (PPE) significantly reduced respiratory symptoms.

The prevalence of chronic symptoms in the current study was consistent with a study conducted in Malaysia, India, and Tanzania, which determined symptom levels of 24.5%, 21.4%, and 21.6% respectively [17–19]. In addition, the prevalence of coughing and breathlessness were similar to studies conducted in India and Iran, which found 15.5% coughing, 3.9% breathlessness, 17.8% coughing, and 2.35% breathlessness respectively [3, 20]. Generally, the elevated prevalence of respiratory symptoms may be attributable to exposure to welding fumes in their work environment. This assertion finds support in a study conducted among welders in Kazakhstan’s metalworking industries, which reported significantly increased levels of FeNO. Elevated FeNO levels are indicative of potential inflammation in the respiratory tract due to airborne exposure at the workplace [9]. 

The finding concerning personal welding fume exposure also showed some similarities with a study conducted in Saudi Arabia in 2010, which showed a mean value of personal welding fume exposure among welders forsix factories 6.23 mg/m^3^ [21]. Another study conducted in 2018 in Dar es Salaam, Tanzania showed that the mean personal welding fume exposure among small-scale welders was 6.57mg/m^3^ [22]. In addition, a study from Tehran, Iran, in 2009, showed the personal total welding fume exposure among welders to be 6.37 mg/m^3^ [19]. However, the present finding is a bit lower than a study conducted in South Korea, and in Saudi Arabia in 2015 where the average welding fume exposure was 7.7 mg/m^3^ and 7.12 mg/m^3^ respectively [6, 23].

In this study, 16 samples (53.3%) surpassed the occupational exposure limit of 5 mg/m3 established by both the American Conference of Governmental Industrial Hygienists (ACGIH) and the Occupational Safety and Health Administration (OSHA) [16]. This aligns with findings from a study in Sweden where 50% of participating welders exceeded the total weighted average exposure limit for welding fumes [24].In the present study, the prevalence of chronic respiratory symptoms was higher among study participants who only attended primary and secondary education than those who attended college education. This finding was in agreement with findings from Tanzania, which showed a higher proportion of respiratory symptoms, with a statistically significant association among welders who attended only lower education [22]. This might be because such welders had a lower awareness of the health effects of welding on respiratory problems.

In this study, welders who were not using respiratory protective equipment (RPE) were more likely to develop chronic respiratory symptoms. This finding was consistent with the study conducted in Tanzania and India among welders indicated that those who did not use RPE properly were more likely to develop respiratory symptoms than those who used such equipment properly [18, 22].

In the present study welders who were not given training on occupational safety were more likely to develop a chronic respiratory symptom than those who did not get training. This might be because the welders utilized RPE more effectively after the training [20].

In this study, the odds of developing a chronic respiratory symptom were higher among welders who did not calibrate or maintain their welding machine properly than those who calibrated the welding machine. This could be related to older welding machines that produced more fumes than the newer ones. This might lead to overexposure to welding fumes and lead to an increased likelihood of developing a chronic respiratory symptom [25].

Compared to welders who did their welding tasks outdoors, the odds of developing chronic respiratory symptoms were higher among welders who worked indoors. This could be related to insufficient air ventilation in the indoor working area [26].

This study was not without limitation, as this study was conducted during the COVID-19 pandemic some of the respondents, both metal and office workers, were afraid to admit that they experiencing respiratory symptoms. This might have resulted in an underestimation of the prevalence of respiratory symptoms. As this study used interviews to assess chronic respiratory symptoms there might be a recall bias. Another limitation of this study was the lack of lung function measurement or account for the baseline medical condition of the welders as well as the absence of a non-ideal choice of a control group. Additionally, the use of a closed-faced cassette during measurements may cause dust particles to adhere to the interior of the cassette, potentially resulting in an underestimation of the exposure level.

Given the high prevalence of respiratory symptoms, the owner of the metalworking establishment should provide metal workers with proper respiratory protective equipment. Additionally, they should establish a schedule for regular welding machine calibrations, maintenance, or replacing older welding machines with new ones. Furthermore, there is a need for a longitudinal study to characterize the association between welding fume exposure and respiratory health that can influence policymakers and improve the health of welders.

## Conclusion

The overall prevalence of chronic respiratory symptoms among welders was higher than controls. The findings indicated that 53.3% of the samples were found to exceed the occupational exposure limit set by the American Conference of Governmental Industrial Hygienists (ACGIH). Safety training, respiratory protective equipment, and welding sites were major factors associated with chronic respiratory symptoms.

## Data Availability

The dataset generated and/or analyzed during the present study is available from the corresponding author.
